# Psychological resilience and active aging among older people with mobility limitations

**DOI:** 10.1007/s10433-020-00569-4

**Published:** 2020-05-04

**Authors:** Sini Siltanen, Anu Tourunen, Milla Saajanaho, Lotta Palmberg, Erja Portegijs, Taina Rantanen

**Affiliations:** Gerontology Research Center, Faculty of Sport and Health Sciences, University of Jyväskylä, PO Box 35, 40014 Jyvaskyla, Finland

**Keywords:** Coping, Adaptation, Walking, Participation, Successful aging

## Abstract

Active aging refers to striving for well-being through preferred activity and may be restricted with declining mobility. We investigated whether psychological resilience, i.e., the ability to tolerate hardship, can aid older people in being active despite mobility limitations. Participants were 961 community-dwelling persons aged 75, 80, or 85 years living in Jyväskylä, Central Finland. Mobility limitations were indicated as self-reported difficulty in walking 2 km. Categories were no difficulty (reference), difficulty, and unable to walk. Resilience was assessed with the 10-item Connor–Davidson Resilience Scale and active aging with the University of Jyvaskyla Active Aging scale. Data were analyzed with OLS regression analyses, which were stratified by age. In all age-groups, having difficulties walking or being unable to walk 2 km was associated with lower active aging scores. Resilience moderated this association especially among the 75-year-olds, but not among the 85-year-olds: The higher the resilience score, the higher the active aging score among those reporting no or some walking difficulties. Those unable to walk 2 km had lower active aging scores irrespective of resilience level. Psychological resilience may alleviate the negative effects of early phase walking difficulties on active aging but may be insufficient to compensate for more severe walking limitations that restrict not only function but also autonomy.

**Electronic supplementary material:**

The online version of this article (10.1007/s10433-020-00569-4) contains supplementary material, which is available to authorized users.

## Introduction

Gerontology no longer views aging solely as a time of disease and disability. Studies have approached aging well through several, somewhat overlapping, concepts such as successful, healthy, or active aging. In the MacArthur model, features of successful aging include maintaining good physical health, good mental and physical function, and active engagement with life (Rowe and Kahn 1987). Although being one of the most ubiquitous models of aging well, it has also been criticized for being too exclusive and correlating poorly with older people’s perceptions of aging well (McLaughlin et al. 2012; Pruchno and Carr 2017; Rowe and Kahn 1987, 2015; Strawbridge et al. 2002). Drawing on the activity and self-determination theories (Havighurst 1961; Ryan and Deci 2000), the World Health Organization (WHO) set out the active aging policy framework (World Health Organization 2002). The framework emphasizes opportunities for participation in activities that correspond to the rights, goals, needs, and capacities of people as they age. It is also more inclusive and considers older people’s own voices. However, since the WHO framework is designed to guide policies and societal actions, researchers have been unable to use it to model individual-level data (Bélanger et al. 2017; Paúl et al. 2012).

Drawing on the WHO framework, we recently defined active aging of individuals as “the striving for elements of wellbeing through activities relating to a person’s goals, functional capacities and opportunities”(Rantanen et al. 2019). Our viewpoint includes activity and ability but also the will to act and overall opportunities for activity. As personal preferences are in a key role, active aging may manifest in various ways. Hence, for empirical purposes, we created and validated a quantitative self-rating scale (Rantanen et al. 2019) that incorporates several essential life areas in line with the categories of Activities and Participation of the International Classification of Functioning, Disability and Health (ICF) (World Health Organization 2001). Higher active aging scores were found to correlate with better quality of life and perceived health and autonomy and with greater life-space mobility (Rantanen et al. 2019), which is an indicator of community mobility and participation. To understand these associations better, the factors influencing and underlying active aging merit further investigation.

Difficulty walking longer distances is a common adversity among older people and typically the first sign of functional decline, which may eventually progress to inability and even difficulty with shorter distances (Mänty et al. 2007; Rantanen 2013; Verbrugge and Jette 1994). Walking difficulties make leaving the home and accessing local amenities and activities more burdensome (Rantakokko et al. 2013; Rantanen 2013). Hence, they may further increase the risk for reduced life-space mobility and eventually lead to the abandonment of valued activities outside the home (Rantakokko et al. 2017). Nevertheless, some older persons are able to remain active and maintain relatively higher level of outdoor mobility despite walking difficulties (Morrow-Howell et al. 2014; Rantakokko et al. 2017) and may thus possess some other, possibly psychological, resources. We recently reported that tenacity and flexibility in goal pursuit are associated with higher life-space mobility and perceived autonomy in participation outdoors (Siltanen et al. 2019) and that tenacious older persons are more likely to engage in leisure time activities in the face of physical decline than less tenacious counterparts (Tourunen et al. 2019a). Tenacity and flexibility relate to psychological resilience, which refers to the ability to adapt positively to adversity and overcome stressful situations (Dyer and McGuinness 1996).

Psychological resilience may enable people to maintain a higher level of activity regardless of walking difficulty and thus contribute to active aging of individuals. Resilience has been characterized as a dynamic process underlying individual differences in response to life hazards (Luthar et al. 2000; Rutter 2006) and as a more stable personal trait manifesting even in the absence of a stressful situation (Luthar et al. 2000). Resilience has also been described by its attributes, such as high self-efficacy in specific tasks and situations (Gillespie et al. 2007; Hicks and Conner 2014). It is commonly agreed, however, that resilience contributes to well-being and quality of life when confronting adversities and hence may be a key resource for aging well. Some previous studies have shown that higher levels of resilience are associated with higher levels of physical activity (Perna et al. 2012; Resnick et al. 2018) and social participation (Levasseur et al. 2017). However, it is unclear whether resilience contributes to active aging among people facing mobility impairments.

This study examined (1) the associations of active aging with difficulties in walking 2 km and psychological resilience, and (2) whether resilience moderates the association between walking difficulties and active aging among community-dwelling people aged 75, 80, and 85 years. We hypothesized that walking difficulties increase the likelihood of lower active aging scores and that higher resilience mitigates this association. We also assumed that such mitigation would likely depend on the severity of walking difficulty and age.

## Methods

### Data and participants

The present analyses form part of the *Active aging*—*resilience and external support as modifiers of the disablement outcome* (AGNES) study. Details of the design and protocol have been reported elsewhere (Rantanen et al. 2018). Briefly, AGNES is a population-based, observational study of three age cohorts (75, 80, and 85 years) conducted at the University of Jyväskylä, Finland. Based on power calculations, a sample size of 1000 persons was needed to provide a 99% power to demonstrate a contribution to the explained variance of 5% in a linear regression model with 10 predictors. A sample of 2791 community-dwelling older persons living in the Jyväskylä area in Central Finland was drawn from the national population register. Of these, 2348 persons were reached and informed about the study. All those willing to participate and able to communicate with the interviewers were included in the study (*N* = 1021) (Portegijs et al. 2019). For this study, data were collected between 2017 and 2018 via computer-assisted, face-to-face interviews in the participants’ homes (*n* = 1018). The present analyses were performed only for participants with data on active aging, walking difficulties, and psychological resilience (*n* = 961). Of these, 557 were women and 404 were men, and 46% were aged 75, 33% aged 80, and 21% aged 85.

All participants signed a written informed consent. The study protocol was approved by the Central Finland Health Care District on August 23, 2017.

### Variables

*Active aging* was assessed using the University of Jyväskylä Active Aging Scale (UJACAS) (Rantanen et al. 2019). The UJACAS scale consists of 17 activity items: practicing memory, using a computer, advancing matters in one’s own life, exercising, enjoying the outdoors, taking care of one’s personal appearance, crafting or DIY, making one’s home cozy and pleasant, helping others, maintaining friendships, getting to know new people, balancing personal finances, making one’s days interesting, practicing artistic hobbies, participating in events, advancing societal/communal matters, and doing things in accord with one’s world view. Each activity is assessed from four perspectives: willingness (to what extent the person wants to do the activity), ability (to what extent the person is able to do it), opportunity (to what extent the person perceives opportunities to do it), and activity (how often or how much the person does it). Respondents were asked to assess each item with respect to the past 4 weeks and give an answer on a five-point Likert scale ranging from 0 (lowest) to 4 (highest). The response options are worded to suit the items, for example from “not at all” to “daily or almost daily” for activity and from “not at all” to “very strongly” for willingness. The scores were summed to form, first, four subscores (willingness, ability, opportunity, activity; range 0–68 in each) and second, for participants with at most two missing items in each subscore, a total score (range 0–272). The following formula was used to impute missing data: (sum score/items responded to) *x* items offered. Higher scores indicate a higher level of active aging. The reliability and validity of the measure are good (Rantanen et al. 2019).

*Walking difficulties* were assessed based on validated self-reports (Mänty et al. 2007). Participants were asked whether they were able to walk 2 km. The response options were “able without difficulty,” “able with some difficulty,” “able with a great deal of difficulty,” “unable without the help of another person,” and “unable to manage even with help.” To reduce the number of dimensions, walking difficulties were recoded into three categories: no difficulty, some or a great deal of difficulty, and unable to walk 2 km (with or without the help of another person). Those reporting no difficulties in walking formed the reference category.

*Psychological resilience* was assessed with a slightly modified, shortened version of the Connor–Davidson Resilience Scale (CD-RISC), which showed good validity and acceptable reliability in the AGNES sample (Tourunen et al. 2019b). Unlike in the original scale, which refers to the previous 4 weeks, we asked the participants to consider their life in general when responding. The shortened scale consists of ten items that reflect the respondent’s ability to tolerate and bounce back from a variety of challenges in life, e.g., “can deal with whatever comes,” “can achieve goals despite obstacles,” and “not easily discouraged by failure” (Campbell-Sills and Stein 2007). The 5-point Likert response scale ranges from not true at all (0) to true nearly all of the time (4). A sum score was calculated (range 0–40, higher scores indicating more resilience) when at least seven items received a response. For the 12 participants who had 1–3 missing items, we imputed new values based on the means of their existing values. In addition, 48 (4.7%) participants had more than three missing items and were not included in the analyses. For sensitivity analysis, resilience was classified into tertiles: highest (≥ 34), middle (33–30), and lowest (≤ 29).

#### *Covariates*

Age, sex, morbidity, education, living alone, and cognitive function were set as covariates, since they correlated with at least one of the predictors and/or outcome (Supplementary Table 1). Age and sex were drawn from the national population register. The number of years of education was self-reported (Rantanen et al. 2019). In line with our previous studies (Rantanen et al. 2012), morbidity was indicated by the number of self-reported physician-diagnosed chronic diseases calculated based on a list of 34 common conditions and an open-ended additional question. Living alone (yes vs. no) was assessed with the question: “Who do you live with?” Global cognitive function was measured with the Mini-Mental State Examination (MMSE) (Folstein et al. 1975).

### Statistical analyses

Participants’ characteristics were described with means and standard deviations (continuous variables) or with percentages (categorical variables). Differences between age-group means or proportions were tested with one-way ANOVA and Bonferroni post hoc test or Chi square test, respectively. To learn whether it would be reasonable to stratify the main analyses by age-group and/or sex, we executed ordinary least squares (OLS) regression analyses in which interactions between age, sex, resilience, and walking difficulties with active aging as outcome were tested. Next, the individual associations of walking difficulties and resilience with active aging were tested with unadjusted OLS models that included only one or the other as an independent variable. Secondly, walking difficulties and resilience were added to the model simultaneously as independent variables. In the final step, walking difficulties and resilience were allowed to interact to test whether resilience moderates the relationship between walking difficulties and active aging. Walking difficulty was coded as multicategorical by using the indicator method. To probe the moderation effect, a pick-a-point approach by regression centering was used with the 16th, 50th, and 84th percentiles of the distribution of the resilience scale describing relatively low, moderate, and relatively high values (Hayes 2017). The moderation analyses were first unadjusted, after which the covariates were added one at a time, and eventually, simultaneously. The analyses were age-stratified.

Finally, as a sensitivity analysis, we tested whether those reporting difficulties walking 2 km in the highest resilience tertile differed in their activity from those reporting difficulties walking 2 km in the lowest resilience tertile. Group differences in the UJACAS activity subscores (continuous variable) were tested with independent samples t test and in the separate activity items (categorical variables) with Chi square test. All analyses were performed with SPSS Statistics 24 for Windows. The PROCESS macro version 3.3 for SPSS was utilized for the moderation analyses (Hayes 2017).

## Results

Table 1 describes the study participants’ characteristics. Active aging scores were lower, and self-reported walking difficulties or inability to walk 2 km was more common among the 85-year-olds than among the younger participants (Table 1). A declining age gradient was observed for length of education, cognitive function, and number of chronic conditions. Living alone was more common among the older participants. Resilience did not differ by age. Those reporting no difficulty walking 2 km had more favorable values in all variables when compared to those reporting difficulties or inability to walk 2 km. In addition, walking difficulties were reported more frequently by women than men.

**Table 1:** Background characteristics of the study participants by age-group and 2 km walking category

	Age				2 km walking difficulties			
	75 yrs *n *= 457	80 yrs *n *= 334	85 yrs *n *= 227		No difficulty *n *= 634	Difficulty *n *= 284	Unable *n *= 76	
	Mean (SD)	Mean (SD)	Mean (SD)	*p*	Mean (SD)	Mean (SD)	Mean (SD)	*p*
Years of education	12.1 (4.2)	11.7 (5.3)	10.2 (4.1)	< .001^a^	11.8 (4.3)	11.5 (5.3)	10.1 (4.3)	.013^a^
Number of chronic conditions	3.2 (2.1)	3.4 (2.0)	3.9 (2.0)	< .001^a^	2.9 (1.7)	4.3 (2.1)	4.8 (2.6)	< .001^a^
Cognitive function (MMSE)	27.6 (2.3)	27.1 (2.7)	26.2 (2.8)	< .001^a^	27.3 (2.4)	27.1 (2.3)	25.4 (3.4)	< .001^a^
Active aging (UJACAS)	200.5 (28.5)	194.0 (31.4)	177.4 (34.0)	< .001^a^	202.3 (26.5)	186.0 (31.1)	150.9 (33.6)	< .001^a^
Resilience (CD-RISC10)	31.4 (5.0)	31.0 (5.2)	30.8 (5.6)	.250^a^	31.6 (4.9)	30.4 (5.5)	29.6 (5.9)	< .001^a^
Women (%)	57.8	55.7	58.6	.760^b^	53.2	66.2	61.8	.001^b^
2 km walking difficulties (%)				< .001^b^	–	–	–	–
No difficulty	73.6	62.5	45.4					
Difficulties	21.1	31.7	39.4					
Unable	5.3	5.8	15.1					
Living alone (%)	33.3	38.9	60.4	< .001^b^	34.4	50.4	63.2	< .001^b^

*SD* standard deviation, *MMSE* mini-mental state examination, *SPPB* short physical performance battery, *CD*-*RISC10* 10-item Connor–Davidson Resilience Scale, *UJACAS* University of Jyvaskyla Active Aging Scale

^a^Tested with one-way ANOVA, ^b^Tested with Chi square

We stratified the main analyses by age-group, as the preliminary OLS analyses indicated that age moderated the associations between resilience and active aging and between walking difficulty and active aging (*p *= 0.005 and *p *= 0.02, respectively). The interactions between sex and walking difficulty and between sex and resilience with the active aging score as outcome were not statistically significant (Supplementary Table 2).

Individually, walking difficulties and resilience accounted for a considerable proportion of the variance of active aging in all three age-groups (Table 2). When compared with those reporting no difficulties in walking 2 km, those reporting walking difficulties or inability to walk 2 km showed lower active aging scores. Higher resilience, in turn, was associated with higher active aging scores. Adding these variables to the model simultaneously did not markedly change the results, except for the 75-year-olds, among whom the relationship between walking difficulties and active aging became nonsignificant.

**Table 2:** The individual associations of 2 km walking difficulties (reporting walking difficulty or being unable to walk independently vs. reporting no difficulty) and resilience (CD-RISC10, range 0–40) with active aging (UJACAS, range 0–272) tested with OLS regression analysis

	Model 1			Model 2			Model 3		
	*B*	95% CI	*R*^2^	*B*	95% CI	*R*^2^	*B*	95% CI	*R*^2^
*75*-*year*-*olds*	*n *= *450*			*n *= *447*			*n *= *445*		
No difficulty	0.0	Ref.	0.19	–	–	–	0.0	Ref.	0.26
Walking difficulty	− 6.38	− 12.27, − 0.49		–	–	–	− 5.01	− 10.62, 0.59	
Unable to walk	− 55.14	− 65.83, − 44.45		–	–	–	− 51.65	− 61.95, − 41.36	
Resilience	−	–	−	1.74	1.24, 2.23	0.10	1.58	1.13, 2.03	0.26
*80*-*year*-*olds*	*n *= *325*			*n *= *323*			*n *= *319*		
No difficulty	0.0	Ref.	0.20	–	–	–	0.0	Ref.	0.39
Walking difficulty	− 19.83	− 26.44, − 13.22		–	–	–	− 17.09	− 22.90, − 11.29	
Unable to walk	− 51.23	− 63.34, − 38.12		–	–	–	− 40.04	− 51.79, − 28.29	
Resilience	–	–	–	3.07	2.50, 3.63	0.26	2.75	2.23, 3.27	0.39
*85*-*year*-*olds*	*n *= *214*			*n *= *199*			*n *= *197*		
No difficulty	0.0	Ref.	0.15	–	–	–	0.0	Ref.	0.33
Walking difficulty	− 15.60	− 24.89, − 6.32		–	–	–	− 10.84	− 19.42, − 2.26	
Unable to walk	− 39.32	− 51.90, − 26.74		–	–	–	− 36.00	− 48.32, − 23.67	
Resilience	–	–	–	2.86	2.11, 3.61	0.22	2.63	1.91, 3.34	0.33

Model 1 included only walking difficulties as an independent variable. Model 2 included only resilience as an independent variable. Model 3 included both walking difficulties and resilience as independent variables. All models were unadjusted. The association is statistically significant if the 95% confidence interval does not include zero

### Moderation effect

**Fig. 1:**
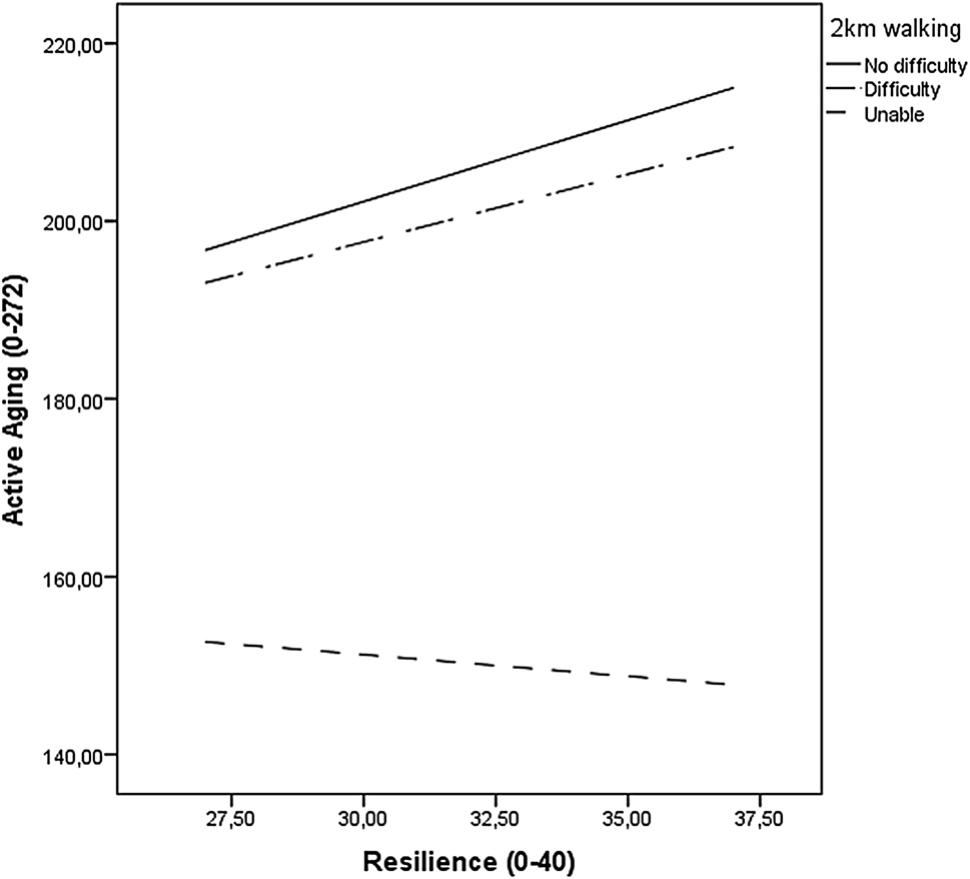
Illustration of the unadjusted OLS path analysis with psychological resilience (CD-RISC10) as a moderator of the relationship between 2 km walking difficulties (reporting walking difficulty or being unable to walk independently vs. reporting no difficulty) and active aging (UJACAS) among the 75-year-olds (n = 445). *Note* Fit for model *R*^2^ = 0.27, *F*(5, 439) = 32.80, *p* < 0.001. The moderation effect was probed using regression centering with the 16th, 50th, and 84th percentiles of the distribution of the resilience scale describing relatively low, moderate, and relatively high values. The effect was significant at all these levels (*p* < 0.001)

Further analysis showed that the interaction of resilience and difficulty walking 2 km with active aging as outcome was significant among the 75- and 80-year-olds. Higher resilience was associated with higher active aging scores for those with no difficulty walking 2 km and for those with difficulty walking 2 km, but not for those reporting inability to walk 2 km (Table 3). The moderation effect was statistically significant at all probed levels of resilience (*p *< 0.001 for all) and remained significant among the 75-year-olds in all the adjusted models. Among the 80-year-olds, the moderation effect was no longer statistically significant in the fully adjusted model, since adjusting for cognitive function attenuated the associations to the point where they became nonsignificant. The unadjusted moderation effects are illustrated in Figs. 1 (75-year-olds) and 2 (80-year-olds). Among the 85-year-olds, the moderation effect was not statistically significant.

**Table 3:** Age-stratified ordinary least squares path analyses with psychological resilience (CD-RISC10, range 0–40) as a moderator of the relationship between 2 km walking difficulties (reporting walking difficulty or being unable to walk independently vs. reporting no difficulty) and active aging (UJACAS, range 0–272)

	Unadjusted model				Fully adjusted model			
	*B*	S.E.	*p*	95% CI	*B*	S.E.	*p*	95% CI
*75*-*year*-*olds*	*n *=* 445*				*n *=* 443*			
No difficulty	0.0	Ref.	Ref.	Ref.	0.0	Ref.	Ref.	Ref.
Walking difficulty	4.48	17.01	0.79	− 28.96, 37.91	12.62	15.93	0.43	− 18.69, 43.94
Unable to walk	18.49	27.06	0.50	− 34.71, 71.68	26.10	25.57	0.31	− 24.17, 76.36
Resilience (Res.)	1.83	0.28	< 0.001	1.28, 2.37	1.90	0.26	< 0.001	1.38, 2.41
No difficulty * Res.	0.0	Ref.	Ref.	Ref.	0.0	Ref.	Ref.	Ref.
Difficulty * Res.	− 0.30	0.54	0.58	− 1.36, 0.76	− 0.48	0.50	0.34	− 1.47, 0.51
Unable to walk* Res.	− 2.32	0.88	0.009	− 4.04, − 0.59	− 2.25	0.83	0.007	− 3.88, − 0.62
*80*-*year*-*olds*	*n *=* 319*				*n *=* 317*			
No difficulty	0.0	Ref.	Ref.	Ref.	0.0	Ref.	Ref.	Ref.
Walking difficulty	− 17.20	17.43	0.32	− 51.29, 17.10	− 22.54	16.44	0.17	− 54.89, 9.82
Unable to walk	41.36	35.68	0.25	− 28.85, 111.58	13.87	33.98	0.68	− 52.99, 80.72
Resilience (Res.)	2.89	0.34	< 0.001	2.22, 3.56	2.66	0.32	< 0.001	2.03, 3.29
No difficulty * Res.	0.0	Ref.	Ref.	Ref.	0.0	Ref.	Ref.	Ref.
Difficulty * Res.	0.01	0.56	0.99	− 1.09, 1.10	0.16	0.53	0.76	− 0.88, 1.20
Unable to walk* Res.	− 2.87	1.23	0.021	− 5.31, − 0.44	− 1.69	1.18	0.15	− 4.01, 0.63
*85*-*year*-*olds*	*n *=* 197*				*n *=* 195*			
No difficulty	0.0	Ref.	Ref.	Ref.	0.0	Ref.	Ref.	Ref.
Walking difficulty	− 29.00	24.97	0.25	− 78.25, 20.25	− 36.62	24.05	0.13	− 84.06, 10.83
Unable to walk	− 9.11	32.42	0.78	− 73.07, 54.84	− 18.23	31.92	0.57	− 81.21, 44.75
Resilience (Res.)	2.53	0.57	< 0.001	1.40, 3.65	2.34	0.55	< 0.001	1.25, 3.42
No difficulty * Res.	0.0	Ref.	Ref.	Ref.	0.0	Ref.	Ref.	Ref.
Difficulty * Res.	0.60	0.80	0.45	− 0.97, 2.17	0.94	0.77	0.22	− 0.57, 2.46
Unable to walk* Res.	− 0.90	1.04	0.39	− 2.94, 1.15	− 0.32	1.02	0.75	− 2.33, 1.68

The fully adjusted model was adjusted for sex, years of education, cognitive function, number of chronic conditions, and living alone

**Fig. 2:**
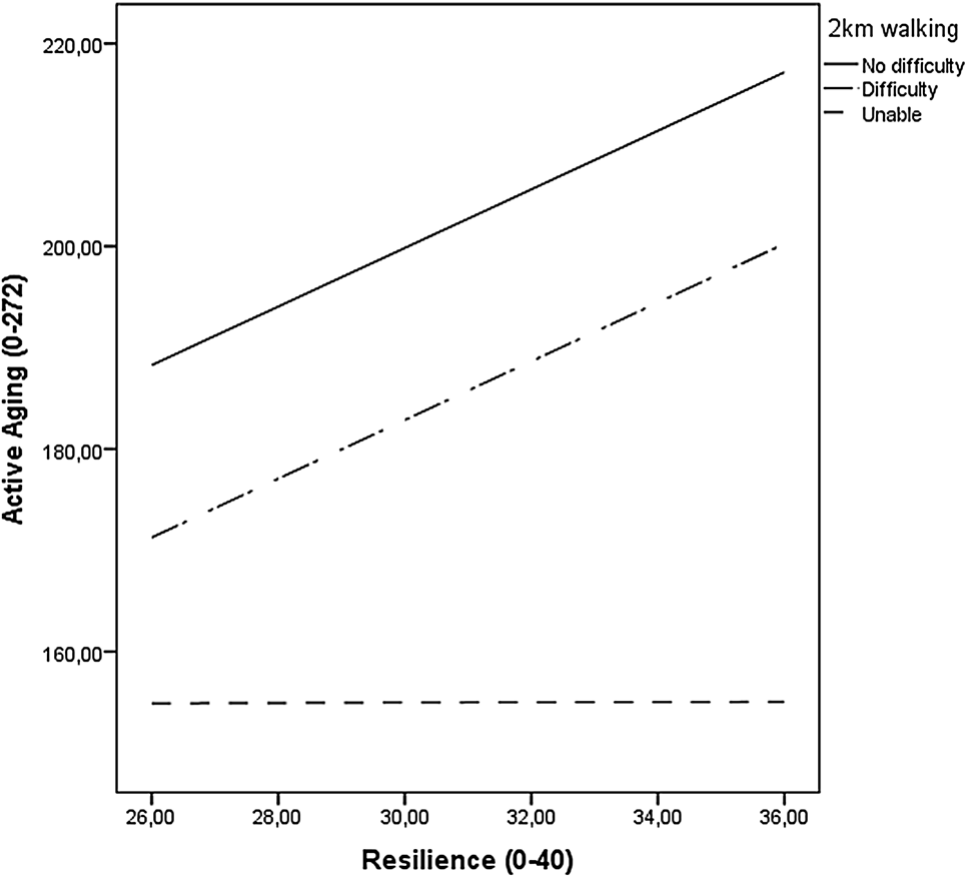
Illustration of the unadjusted OLS path analysis with psychological resilience (CD-RISC10) as a moderator of the relationship between 2 km walking difficulties (reporting walking difficulty or being unable to walk independently vs. reporting no difficulty) and active aging (UJACAS) among the 80-year-olds (n = 319). *Note* Fit for model *R*^2^ = 0.41, *F*(5, 313) = 42.78, *p* < 0.001. The moderation effect was probed using regression centering with the 16th, 50th, and 84th percentiles of the distribution of the resilience scale describing relatively low, moderate, and relatively high values. The effect was significant at all these levels (*p *< 0.001)

### Sensitivity analysis

Those reporting difficulties walking 2 km in the highest resilience tertile (*n *= 88) had higher UJACAS activity subscores than those reporting difficulties walking 2 km in the lowest resilience tertile (*n* = 115; mean 43.4, SD 7.9 vs. *M* 34.8, SD 9.0, respectively, *p *< 0.001). In addition, those with higher resilience were more active, e.g., in practicing memory, advancing matters in one’s own life, making one’s home cozy and pleasant, maintaining friendships, balancing personal finances, making one’s days interesting, and doing things in accord with one’s world view (*χ*^2^ *p* ≤ 0.001) than those in the lowest resilience tertile and reporting difficulties walking 2 km. In contrast, the groups showed no differences in participation in events, helping others or exercising.

## Discussion

These findings establish a novel approach to research on aging well. To our knowledge, this is the first study to show that higher psychological resilience may contribute to active aging among persons with early phase mobility limitations. However, it seems that while psychological resilience may compensate for the negative impact of declining walking ability in its early phase, it no longer has such mitigating effect in the more severe phase of mobility decline.

Information on the association of psychological resilience with functioning of older people has been rather limited. It was only recently found that, despite mobility limitations, older people with high tenacity are more likely to participate in leisure activities (Tourunen et al. 2019a, b) and that those with high tenacity and flexibility are more likely to maintain higher extent and autonomy in outdoor mobility (Siltanen et al. 2019). Tenacity and flexibility in goal pursuit may be considered attributes of resilience. In addition, an earlier study reported that higher levels of resilience prevented ADL and IADL disability at the onset of a new chronic condition (Manning et al. 2014). The present findings are the first to show the importance of resilience for active aging measured with a novel instrument incorporating will, ability, and opportunity to act, and the level of activity in 17 activities describing active agency in essential life areas (Rantanen et al. 2018).

Difficulty in walking longer distances is one of the most common adversities that older people face. In our sample, over one-third overall and over half of the 85-year-olds reported at least some difficulties in walking 2 km. The present findings suggest that resilience may mitigate the negative effects of walking difficulties on active aging. An important aspect of resilience is being determined and persistent in one’s personal aspirations (Lamond et al. 2008) and possibly coming up with new ways to attain them. This may be a plausible explanation for our finding. For example, persons with high resilience may compensate for their mobility limitations by applying adaptive strategies (e.g., assistive devices, slowing down the pace of walking, taking rest breaks), which have been shown to help maintain greater life-space mobility and autonomy in participation in out-of-home activities (Skantz et al. 2020). Active use of compensatory strategies among persons with reduced physical function and high well-being has also been reported elsewhere (Carpentieri et al. 2017).

Another possible explanation for the finding that resilience supports active aging among people at the early phase of mobility decline may be that we studied a comprehensive range of essential life activities. Our idea was to include items that cover a variety of activities of older people and are, in principle, also feasible for people with disabilities (Rantanen et al. 2019). Thus, the items do not have strict objective criteria but are worded to allow the participants themselves to define what the activity involves. For example, for some people the item “maintaining friendships” may mean joint walks and coffee dates, while for others it may mean a phone call. People with walking difficulties and high resilience may strive to engage in their valued activities in ways that better correspond to their declined mobility. For example, they may increase their participation in activities closer to home that make less demands on mobility, rather than giving up on them completely. Our sensitivity analyses showed that these persons were more active, e.g., in practicing memory, balancing finances, making one’s home cozy and maintaining friendships, i.e., in activities that can be performed at home even in the presence of physical limitations.

The findings also indicated that in cases of more advanced mobility limitations, operationalized in this study as the inability to walk 2 km, resilience might lose its mitigating effect. Mobility decline often co-occurs with other functional and health deficits, such as cognitive decline (Atkinson et al. 2005; Clouston et al. 2013; Demnitz et al. 2016) and depression (Milaneschi and Penninx 2014; Thorpe Jr et al. 2011), which may further lessen a person’s striving to engage in various important life areas. Here, those unable to walk 2 km had significantly lower MMSE scores than those in the other walking categories. In addition, being unable to walk 2 km independently may be experienced as such a drastic loss of function, and especially of autonomy, that positive psychological adaptation alone cannot compensate for it. Compared to persons with some walking difficulties, those unable to walk have fewer personal resources and are more dependent on help and support from others (Rantanen 2013). In our sample, those unable to walk 2 km were also most likely to have trouble walking 500 m. Lastly, the resilience scores clustered toward the lower end of the scale among persons reporting inability to walk 2 km. Thus, testing the moderation effect with relatively high values for resilience might have made it hard to detect a significant association as, in reality, only a few participants unable to walk 2 km demonstrated a very high level of resilience.

Finally, we observed no difference between the three age-groups in resilience. Typically, decline in health and functioning accelerates after age 60, and many major changes take place after age 80 (Ferrucci et al. 2016). However, the present finding indicates that, unlike many other personal resources, resilience does not decline with advancing age. Earlier studies have also found that psychological resilience is as high or even higher in older than in young or middle-aged persons (Gooding et al. 2012; Hamarat et al. 2002). This finding supports suggestions that resilience is an essential factor for adapting to aging and for aging well (Hayman et al. 2017).

The roles and functions of resilience may, however, differ in different stages of old age. In this study, resilience was associated with active aging among all age-groups, even when controlling for walking difficulties, yet the moderation effect was robust only among the 75-year-olds. As discussed by Hayman et al. (2017), the oldest-old with high resilience tend to shift their focus onto what they still can rather than cannot do when they confront age-related adversities. Hence, in line with the model of selection, optimization and compensation (Baltes 1997), the oldest-old may focus on selecting and retaining the most meaningful of their activities when facing mobility decline, while the younger-old may be more active in creating compensatory strategies that enable them to continue various activities. Successful compensation requires sufficient resources (Ebner et al. 2006; Saajanaho et al. 2016). This may render the cost–benefit ratio of compensatory efforts unfavorable or compensatory activities no longer possible for the oldest old (Baltes and Smith 2003; Rothermund and Brandstädter 2003), who may lack socioeconomic resources and not see it as realistic to strive for high activity compared to the young-old (Saajanaho et al. 2016). After all, loss-based selection, i.e., focusing on fewer goals, may result in an equally meaningful life and positive adaptation among the 85-year-olds but manifest as lower active aging scores. Other explanations for the nonsignificant moderation effect may be the smaller sample sizes among the oldest-old and early phase cognitive decline, which attenuated the associations among the 80-year-olds.

### Strengths of the study

This study lays a foundation for new, more comprehensive and interdisciplinary hypotheses on the factors underlying active aging. The present approach to aging well also applies to individuals with functional limitations and disability and considers older people’s own preferences. In addition, the present 17-item measure of active aging may capture the phenomenon of active aging in its various forms as older people may be equally *active* but perform very diverse activities. Moreover, this study was population-based and included a large sample of men and women within the age range most vulnerable to functional decline. Further strengths of the study were the utilization of a novel measure of active aging and reporting on a topic with limited prior data.

### Study limitations

The present cross-sectional design does not allow the investigation of causation. Thus, we cannot be certain which factors are predictors and which are outcomes, and whether active aging can be influenced by promoting resilience and mobility. These are issues that await future studies. Another limitation of the study is that, while most likely applicable to western cultures, our findings are not necessarily applicable to other cultures and populations, as resilience may be culture-specific (Tourunen et al. 2019a, b). Moreover, the participants’ resilience scores were rather high in general, and hence, our findings may underestimate the effects of resilience on active aging.

### Conclusions

High levels of psychological resilience may alleviate the negative effects of walking difficulties on active aging. However, high levels of resilience may not fully compensate for more severe impairment in walking ability. Future studies should continue this work by addressing the longitudinal and causal associations between mobility decline, resilience, and active aging to find out whether promoting resilience and mobility would also enhance active aging.

## Electronic supplementary material

Below is the link to the electronic supplementary material.

Supplementary material 1 (PDF 24 kb)
