# Supplementary material for: Psychological resilience and active aging among older people with mobility limitations
Source: Eur J Ageing. 2020 May 4;18(1):65–74. doi: 10.1007/s10433-020-00569-4 (PMC7925737; doi:10.1007/s10433-020-00569-4)
Supplement: Supplementary file 1 — Supplementary material 1 (PDF 24 kb) [file 10433_2020_569_MOESM1_ESM.pdf]

Psychological resilience and active aging among older people with mobility decline

in European Journal of Ageing

Sini Siltanen[1], Anu Tourunen, Milla Saajanaho, Lotta Palmberg, Erja Portegijs, Taina Rantanen

[1] Gerontology Research Center, Faculty of Sport and Health Sciences, University of Jyväskylä, Finland

sini.k.t.siltanen@jyu.fi

Supplementary Table 1. Correlates of active aging (UJACAS 0-272), 2km walking difficulties (no difficulties, difficulties, unable to walk), and resilience (CD-RISC10, 0-40).

	Active aging		Walking difficulty		Resilience	
	N	r	N	r	N	r
Covariates						
Years of education	996	.22[a]	990	-.10	964	.03[a] *ns*
Cognitive function	1003	.34[a]	988	-.14	965	-.03[a] *ns*
Age	1010	-.27[a]	994	.22	970	-.06[a] *ns*
No. of chronic conditions	1009	-.24[a]	993	.36	969	-.12[a]
Sex	1010	.03 *ns*	994	.11	970	-.05 *ns*
Living alone	1010	.14	994	-.19	970	.03 *ns*
Main variables						
Walking difficulties	989	-.36	-	-	961	-.12
Resilience	969	.42[a]	961	-.12	-	-

[a] Tested with Pearson's correlation. Other correlations describe Spearman's rho.

ns = non-significant. Statistical significance of other correlations was $p \leq .001$.

Supplementary Table 2. Ordinary least squares path analyses with sex and age as moderators of the relationship between resilience or 2km walking difficulties and active aging (UJACAS, range 0-272).

	N	B	S.E.	p	95% CI
Age * Resilience	969	.13	.05	.005	.04, .22
Age * No difficulty	989	.00	ref.	ref.	ref.
Age * Walking difficulty		-1.30	.56	.02	-2.40, -.21
Age * Inability to walk		1.44	.87	.10	-.27, 3.15
Sex * Resilience	969	-.48	.36	.19	-1.19, .24
Sex * No difficulty	989	.00	ref.	ref.	ref.
Sex * Walking difficulty		4.26	4.24	.32	-4.07, 12.59
Sex * Inability to walk		-2.88	7.10	.69	-16.80, 11.05
